# Prophylactic accessory renal artery coil embolization for prevention of type II endoleak following endovascular aneurysm repair: a case report

**DOI:** 10.1186/s40792-017-0334-y

**Published:** 2017-04-27

**Authors:** Ryosuke Nishie, Naoki Toya, Soichiro Fukushima, Eisaku Ito, Yuri Murakami, Tadashi Akiba, Takao Ohki

**Affiliations:** 1 Division of Vascular Surgery, The Jikei University Kashiwa Hospital, Kashiwa, Japan; 2 Department of Surgery, The Jikei University Kashiwa Hospital, Kashiwa, Japan; 3 Department of Vascular Surgery, The Jikei University School of Medicine, Tokyo, Japan; 4 Department of Surgery, Division of Vascular Surgery, Jikei University Kashiwa Hospital, 163-1, Kashiwa-shita, Kashiwa, Chiba 277-8567 Japan

**Keywords:** Accessory renal artery, Endovascular aneurysm repair, Abdominal aortic aneurysm

## Abstract

**Background:**

Prior reports indicate that intentional coverage of the accessory renal arteries (ARAs) with a diameter larger than 3 mm during endovascular aneurysm repair (EVAR) increases risk of additional treatment for type II endoleak. Here, we report a case of prophylactic coil embolization for a 4 mm ARA originating from an abdominal aortic aneurysm.

**Case presentation:**

A 76-year-old woman was admitted to our hospital after noticing an abdominal pulsatile mass. Computed tomography (CT) imaging revealed an abdominal aortic aneurysm (AAA) with a maximum diameter of 53 mm. Preoperative CT scan showed a right ARA, 4 mm in diameter, which was considered likely to lead to type II endoleak following EVAR. ARA coil embolization was performed at the time of EVAR. We observed no endoleaks and no infarct of the inferior pole of the right kidney on completion angiography. The postoperative course was uneventful, and the patient was discharged 7 days later. Postoperative eGFR (58.4 ml/min) was not significantly different from preoperative level (56.7 ml/min). After EVAR, blood pressure was under control, and no additional anti-hypertensive medicines were required. Postoperative enhanced CT image showed that the distal portion of the ARA was well perfused without type II endoleak from ARA.

**Conclusions:**

Prophylactic coil embolization for a large ARA originating from an abdominal aortic aneurysm appears to be safe and effective in preventing type II endoleak following EVAR.

## Background

The reported prevalence of accessory renal artery (ARA) is 12 to 49% [1, 2]. Prior reports indicate that kidney function is not affected by ARA coverage during endovascular aneurysm repair (EVAR), though if ARA diameter is larger than 3 mm, additional treatments for type II endoleak are needed [3–6]. Here, we report a case of prophylactic coil embolization for a 4 mm ARA originating from an abdominal aortic aneurysm.

## Case presentation

**Fig. 1:**
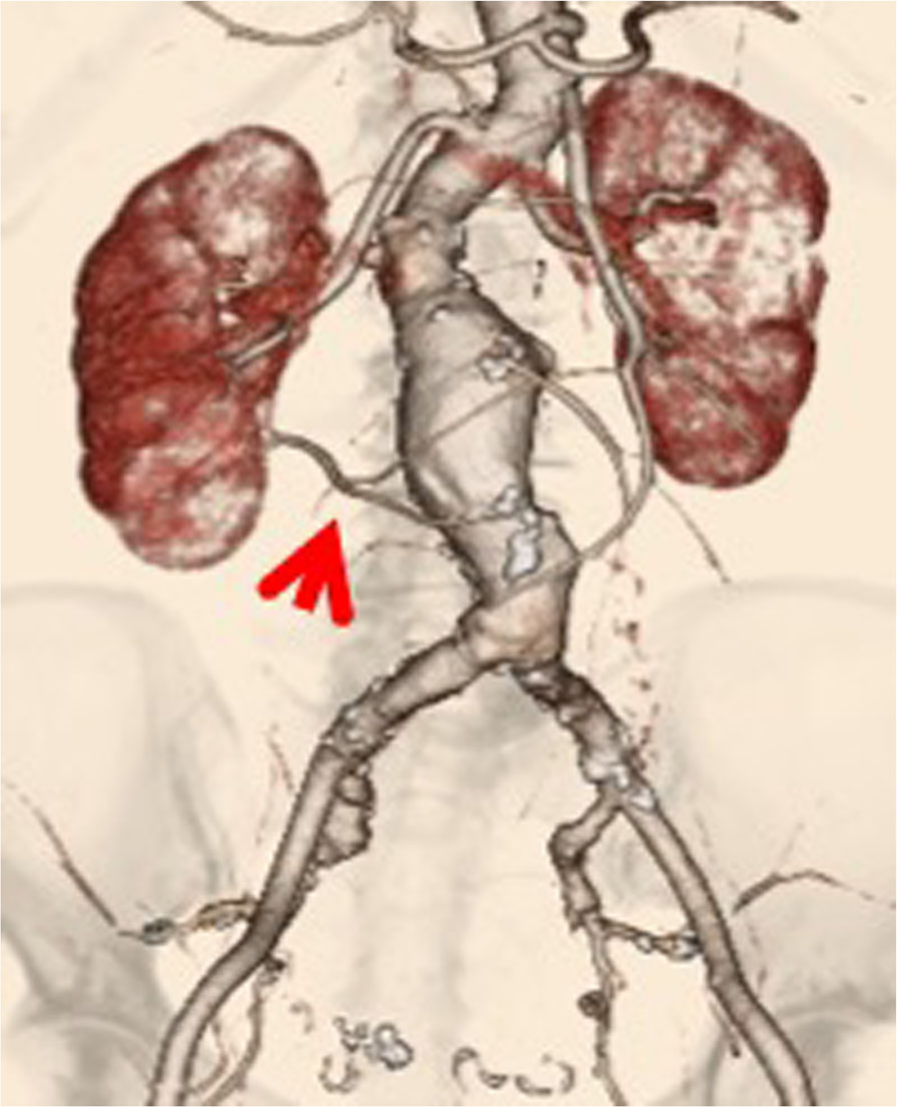
Preoperative enhanced CT. The maximum diameter of the AAA was 53 × 57 mm, and the diameter of the right ARA was 4 mm (*arrow*)

A 76-year-old woman presented at our department with an abdominal pulsatile mass. She had hypertension (nifedipine 20 mg, candesartan 4 mg, indapamide 0.5 mg), dyslipidemia, congestive heart failure, and complete atrioventricular block. She had previously undergone an operation that included endovascular aneurysm repair and prophylactic accessory renal artery coil embolization for advanced uterine cancer. Enhanced computed tomography (CT) imaging revealed an abdominal aortic aneurysm (AAA) with a maximum diameter of 53 × 57 mm and a right ARA (4 mm in diameter) (Fig. 1). Since open repair was considered risky due to her coexisting diseases, we decided to perform EVAR despite the presence of ARA.

**Fig. 2:**
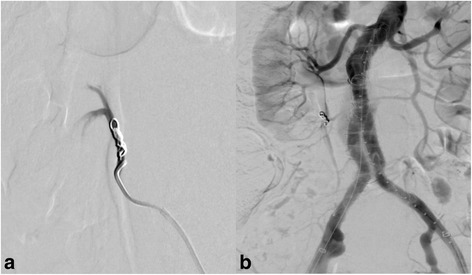
Angiogram during operation. **a** The right ARA was embolized using a coil. **b** The final aortogram showed that the right kidney was well perfused

Under general anesthesia, bilateral transfemoral access was obtained via surgical cutdown, and 7Fr short sheaths were placed in both common femoral arteries. Prior to EVAR, the ARA was selectively catheterized with a 6Fr RDC guiding catheter and a 5Fr RDC catheter, and embolization was performed with two 0.018-in coils (Interlock, Boston Scientific, Marlborough, MA, USA). After coiling, the main body of the stentgraft (Aorfix™, Lombard Medical, Oxfordshire, UK) was deployed with its proximal end just below the renal arteries, and bilateral common iliac arteries were used as distal landing zones. We completed the procedures having seen no perfusion defect of the inferior pole of the right kidney from the right renal artery on completion angiography (Fig. 2).

**Fig. 3:**
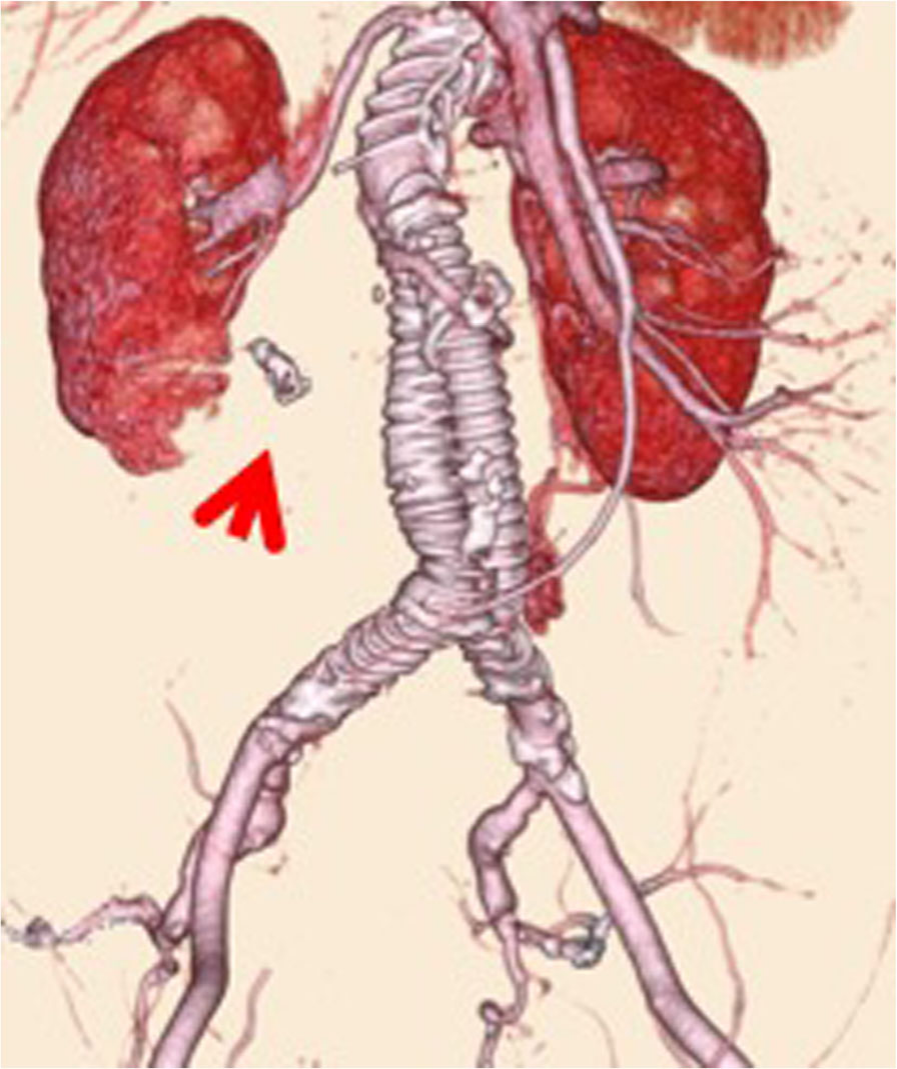
Post-operative enhanced CT. There was no type II endoleak from the ARA. The distal portion of the ARA from the coils was well perfused (*arrow*) and the right kidney was not infarcted

The postoperative course was uneventful, and the patient was discharged 7 days later. Postoperative eGFR (58.4 ml/min) was not significantly different from preoperative (56.7 ml/min). After surgery, blood pressure was under control, and no additional blood pressure medicines were required. Postoperative enhanced CT showed that there was no endoleak. The distal portion of her right ARA from coils including the right kidney was well perfused (Fig. 3).

## Discussion

The reported prevalence of ARA ranges from 12 to 49% [1, 2]. The problems most likely to occur during EVAR for AAA with ARA are renal infarction caused by ARA obstruction and type II endoleaks from ARA blood flow. As ARA coverage has been reported to increase risk of renal infarction, with reported rates ranging from 0 to 84%, ARA reconstruction procedures have been devised using fenestrated stentgrafts, branched stentgrafts, or debranching [7–9]. Yet some believe that the reconstruction of ARA is unnecessary since it offers no significant benefit in terms of eGFR or blood pressure control [3–6]. Type II endoleak occurs from the lumbar artery, inferior mesenteric artery (IMA), median sacral artery, and accessory renal artery (ARA). These branches act as inflow or outflow depending on the pressure gradient between the aorta and each branch. When a renal artery is covered with a stentgraft, the pressure on the kidney parenchyma is decreased. Yet covering the ARA while leaving the renal artery uncovered would result in high pressure, and the ARA would act as inflow. Therefore, ARA larger than 3 mm has been reported as a potential cause of type II endoleak [5]. Because of this, prophylactic ARA coil embolization for ARAs larger than 3 mm is effective for reduction of type II endoleak. In general, routine embolization for lumbar artery, IMA, and median sacral artery is not recommended. In our case, we did not embolize any of these branches.

No previous report has discussed the differences between ARA arising from the aortic neck and that arising from the aneurysm wall. In our case, because the ARA arose from the aneurysm wall and was over 4 mm in diameter, the patient was considered at high risk for type II endoleak from ARA; accordingly, we decided to perform the ARA coil embolization. After coiling, contrary to our concerns, eGFR and blood pressure were well under control, and no type II endoleak from ARA was seen. Additional studies are required to further decrease the incidence of type II endoleaks.

## Conclusions

Prophylactic coil embolization for a large ARA originating from an abdominal aortic aneurysm appears to be safe and effective in preventing type II endoleak following EVAR.

## Abbreviations

AAA: Abdominal aortic aneurysm
ARA: Accessory renal artery
EVAR: Endovascular aneurysm repair
